# Reduction in the incidence of diabetes lower extremity amputations in Queensland: 2005-2010

**DOI:** 10.1186/1757-1146-6-S1-O20

**Published:** 2013-05-31

**Authors:** Peter A Lazzarini, Sharon R O’Rourke, Anthony W Russell, Patrick H Derhy, Maarten C Kamp

**Affiliations:** 1 Allied Health Research Collaborative, Metro North Hospital & Health Service, Queensland Health, Brisbane, Queensland, 4032, Australia; 2 Department of Podiatry, Metro North Hospital & Health Service, Queensland Health, Brisbane, Queensland, 4032, Australia; 3 School of Clinical Sciences, Queensland University of Technology, Brisbane, Queensland, 4059, Australia; 4 Cairns Diabetes Centre, Queensland Health, Cairns, Queensland, 4870, Australia; 5 Department of Diabetes & Endocrinology, Princess Alexandra Hospital, Brisbane, Queensland, 4012, Australia; 6 Diamantina Institute, The University of Queensland, Brisbane, Queensland, 4072, Australia; 7 Centre for Healthcare Improvement, Queensland Health, Brisbane, Queensland, 4029, Australia; 8 School of Medicine, The University of Queensland, Brisbane, Queensland, 4072, Australia; 9 Department of Endocrinology, Metro North Hospital & Health Service, Queensland Health, Brisbane, Queensland, 4029, Australia

## Background

Lower extremity amputation is a common end stage complication among people with diabetes. Since 2006, the Queensland Diabetes Clinical Network has implemented programs aimed at reducing diabetes-related amputations. The aim of this retrospective observational study was to determine the incidence of diabetes lower extremity amputations in Queensland from 2005 to 2010.

## Methods

Data on all Queensland diabetes-related lower extremity amputation admissions from 2005-2010 was obtained using diabetes amputation-related ICD-10-AM (hospital discharge) codes. Queensland diabetes amputation incidences were calculated for both general and diabetes populations using population data from the Australian Bureau of Statistics and National Diabetes Services Scheme respectively. Chi-squared tests were used to assess changes in amputation incidence over time.

## Results

Overall, 4,443 admissions for diabetes-related amputation occurred; 32% (1,434) were major amputations. The diabetes-related amputation incidence among the general population (per 100,000) reduced by 18% (18.2 in 2005, to 15.0 in 2010, *p* < 0.001); major amputations decreased by 24% (6.6 to 4.7, *p* < 0.01). The incidence among the diabetes population (per 1,000) reduced by 40% (6.7 in 2005, to 4.0 in 2010, *p* < 0.001); major amputations decreased by 45% (2.3 to 1.2, *p* < 0.001).

## Conclusion

This paper appears to be the first to report a significant reduction in diabetes amputation incidence in an Australian state. This decrease has coincided with the implementation of several diabetes foot clinical programs throughout Queensland. Whilst these results are encouraging in the Australian context, further efforts are required to decrease to levels reported internationally.

